# An asymmetric PCR-based, reliable and rapid single-tube native DNA engineering strategy

**DOI:** 10.1186/1472-6750-12-39

**Published:** 2012-07-06

**Authors:** Yanzhen Bi, Xianfeng Qiao, Zaidong Hua, Liping Zhang, Ximei Liu, Li Li, Wenjun Hua, Hongwei Xiao, Jingrong Zhou, Qingxin Wei, Xinmin Zheng

**Affiliations:** 1 Hubei Key Laboratory of Animal Embryo Engineering and Molecular Breeding, Institute of Animal Science and Veterinary Medicine, Hubei Academy of Agricultural Science, Wuhan, 430064, China

**Keywords:** Asymmetric, Bridge PCR, Intramolecular homologous recombination, Myostatin

## Abstract

**Background:**

Widely used restriction-dependent cloning methods are labour-intensive and time-consuming, while several types of ligase-independent cloning approaches have inherent limitations. A rapid and reliable method of cloning native DNA sequences into desired plasmids are highly desired.

**Results:**

This paper introduces ABI-REC, a novel strategy combining asymmetric bridge PCR with intramolecular homologous recombination in bacteria for native DNA cloning. ABI-REC was developed to precisely clone inserts into defined location in a directional manner within recipient plasmids. It featured an asymmetric 3-primer PCR performed in a single tube that could robustly amplify a chimeric insert-plasmid DNA sequence with homologous arms at both ends. Intramolecular homologous recombination occurred to the chimera when it was transformed into *E.coli* and produced the desired recombinant plasmids with high efficiency and fidelity. It is rapid, and does not involve any operational nucleotides. We proved the reliability of ABI-REC using a double-resistance reporter assay, and investigated the effects of homology and insert length upon its efficiency. We found that 15 bp homology was sufficient to initiate recombination, while 25 bp homology had the highest cloning efficiency. Inserts up to 4 kb in size could be cloned by this method. The utility and advantages of ABI-REC were demonstrated through a series of pig myostatin (MSTN) promoter and terminator reporter plasmids, whose transcriptional activity was assessed in mammalian cells. We finally used ABI-REC to construct a pig MSTN promoter-terminator cassette reporter and showed that it could work coordinately to express EGFP.

**Conclusions:**

ABI-REC has the following advantages: (i) rapid and highly efficient; (ii) native DNA cloning without introduction of extra bases; (iii) restriction-free; (iv) easy positioning of directional and site-specific recombination owing to formulated primer design. ABI-REC is a novel approach to DNA engineering and gene functional analysis.

## Background

Currently, there are two options available with respect to the creation of recombinant plasmids for gene regulation analysis. These are ligation-dependent and ligation-independent cloning (LDC and LIC). LDC, which includes TA cloning and heterostagger cloning, makes use of restriction enzyme digestion and DNA ligation to produce the desired recombinants. The disadvantages of LDC are that it is time-consuming and labor-intensive, and that the availability of restriction sites is a rate-limiting step, especially for long sequences of interest. In addition, the cloning efficiency of LDC varies. It is largely dependent on the efficacy of restriction cutting and DNA ligation. LDC can also introduce undesired operational sequences such as restriction sites at the junction of functional modules [1]. To circumvent these limitations, several types of LIC methods were developed. Uracil DNA glycosylase (UDG) selectively degrades dUMP residues at the termini of PCR products, which has been incorporated into primers in advance, and generates 3’ overhangs. The processed products can be directionally cloned by annealing with vectors that were complementary to these 3’ overhangs, without *in vitro* ligation [2,3]. Recombinase-dependent cloning utilizes recombinase as the cloning enzyme to catalyze the fusion of the insert into the target vector with homologous ends, but it is generally provided as proprietary components of commercial kits, which can be costly [4,5]. PCR-mediated cloning methods usually rely on so-called megaprimer to produce the desired hybrid sequences, but the efficiency of mega-extension is frequently variable, requiring significant labor input to establish optimal conditions [6,7]. A rapid and reliable method of cloning target DNA is keenly desired.

In gene functional studies and transgene biology, there has been an increasing need to leave no any heterogenous nucleotides within expression plasmids [8]. Typically, common cloning methods will result in inclusion of extra sequences like restriction endonuclease sites or plasmid polylinker sequences, which may be as much as one hundred nucleotides in length. These sequences can change the spacing between DNA elements, which can have undesirable effects on the structure and activity of fusion protein and interfere with accurate analysis. They must be found to be free of translational start or stop sites. In certain cases, they must be examined to ensure the absence of unwanted functional elements. These limitations will compromise the applications of recombinant plasmids that contain extraneous residues [9]. A seamless cloning method that would guarantee that only intact DNA sequences have been manipulated and assembled is highly desired.

In this paper, we outline a rapid and reliable DNA cloning approach, combining asymmetric single-tube bridge PCR with intramolecular homologous recombination in bacteria to create plasmids without any extraneous nucleotides. We first conducted a proof-of-concept study by using a double-resistance reporter system to prove that this novel method yielded expected recombinants with 100% efficiency and fidelity. Effects of homology and insert length upon its cloning efficiency were then investigated. Next, we used this method to clone the regulatory elements of the porcine myostatin gene (MSTN) into luciferase reporters and assessed their expressivity in cultured mammalian cells. Finally we showed that the identified porcine MSTN regulatory elements could coordinately drive the expression of EGFP when organized as an expression cassette. This method was found to be reliable, efficient and site-specific. We believe that it will be widely applicable in DNA engineering and gene functional studies.

## Results

### Validation of ABI-REC through double-resistance reporter assay

**Figure 1:**
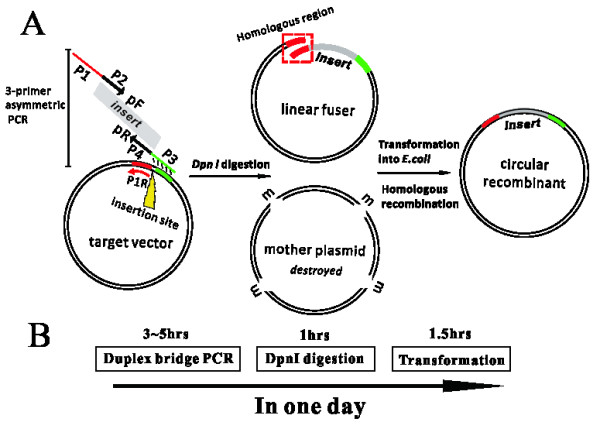
**Concept of ABI-REC cloning.** (**A**) A brief description of the principle of ABI-REC. For details, please refer to text (Results). The green rectangle represents the fusion bridge. The red rectangle represents the upstream homolog arm. The grey rectangle represents the insert of interest. The yellow triangle represents the pre-selected fusion site. The lowercase m represents methylated nucleotides within the plasmid. (**B**) Time schematic. ABI-REC allows the rapid creation of recombinant plasmids within one day.

In a systematic study of pig muscle-specific gene regulatory domains, we planned to clone several DNA sequences for an assessment of transcriptional activity. However, the unavailability of restriction endonuclease sites rendered the cloning of these long sequences too slow by common ligase-dependent methods. The site selection, digestion and ligation processes consumed too much time and labor. We then decided to develop a novel cloning method independent of restriction site and ligase. Inspired by the principle of Quickchange site-directed mutagenesis (Stratagene, La Jolla, CA, U.S.), we propose that a long sequence of interest could be inserted into a specific site of target plasmid in a ligase-independent manner. The concept is shown in Figure 1. In the first step, a bridge PCR is performed to produce a long fragment fusing the insert of choice with the desired vector. Three primers are required for a successful bridge PCR (for primer design, please refer to “methods”). In the primary reaction, pF and pR primers are used to amplify the insert of choice. In the secondary reaction, the resulting insert of choice would anneal with the homologous sequence in the target plasmid for extension to generate the long fused fragment. The ternary process proceeds with pF and P1R primers to amplify the large fragment in an exponential manner. The linear fusion fragment bears homologous ends at 5′ and 3′ ends. Second, the PCR products are digested by DpnI to degrade methylated circular plasmids. Thirdly, the digested products are transformed into *E.coli* to generate the circular fusion plasmid. The identities of these recombinants can be verified by sequencing.

**Figure 2:**
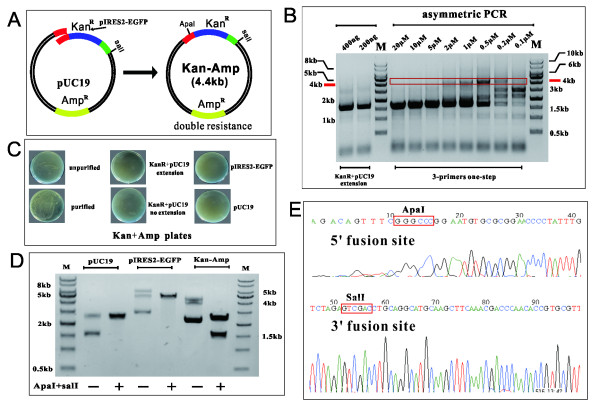
**Validation of ABI-REC through double-resistance reporter assay.** (**A**) The design of double resistance reporter assay. The fusion of the 1.6 kb kan^R^ cassette (from pIRES2-EGFP) with the 2.7 kb pUC19 plasmid (ampicillin resistance) renders the new recombinant plasmid resistant to both kanamycin and ampicillin. The plasmid grows on (Kan + Amp) LB plates. An artificial ApaI restriction site is introduced into the 5′ fusion site to precisely localize the insertion site. (**B**) An asymmetric bridge PCR efficiently fuses the Kan^R^ cassette into the pUC19 plasmid and generates a hybrid fragment. The gradient concentration of the primer pR was assessed and identified as within the optimal range from 2 nM to 0.2 nM in the bridge PCR. A conventional two-primer PCR reaction was conducted in parallel to compare their amplification efficiency. This indicates that the quantity of pR primer is critical to the output of fused sequence in the bridge PCR reaction. (**C**) Double resistant colonies were found to grow on (Kan + Amp) LB plates. Bridge PCR products, purified fused sequences, two-primer PCR products (extension), and a mixture of purified Kan^R^ cassettes with pUC19 (no extension) were transformed into DH5α competent cells. Double resistant colonies were present for bridge PCR products and purified fused sequence, whereas the latter two did not produce viable colonies. This implies that intramolecular recombination occurs within the fused sequence, producing double resistant plasmids that renders cells resistant to both kanamycin and ampicillin. pUC19 and pIRES2-EGFP plasmids were not able to grow on the double-drug plates, precluding the risk of random integration of Kan^R^ cassette into the *E.coli* genome. (**D**) Plasmids of the single colonies in (Kan + Amp) LB plates were extracted and digested using SalI and ApaI. As shown in the electrophoresis gel, the 1.6 kb insert was released, indicating that the Kan^R^ cassette had been fused into pUC19 at pre-determined site. (**E**) Sequencing of the single colonies revealed the insertion of the Kan^R^ cassette and the presence of artificial ApaI restriction site, further proving that the insert of choice has been fused with target plasmid in a restriction- and ligase-free manner. The red rectangle represents the restriction sites.

We designed a double-resistance reporter assay as a proof-of-principle experiment by inserting the kan^R^ cassette of pIRES2-EGFP into an ampicillin-resistant pUC19 plasmid. As illustrated in Figure 2A, when the kan^R^ cassette was fused with pUC19, the resulting vector was resistant to both kanamycin and ampicillin. We attempted to obtain the fused sequence in one turnaround of a single-tube PCR reaction. We first began by titrating the concentration of pR primer from 20 μM to 0.1 μM due to the following considerations: (1) to control the amount of insert in an suitable level to anneal with pUC19 and generate linear sequences and (2) to permit KOD Plus polymerase to focus on amplifying the linear fragment in an exponential manner. It was found that an asymmetric PCR with pR primer from 2 μM to 0.2 μM would produce readily detectable quantities of linear sequence (4.4 kb, red rectangle; Figure 2B). In comparison, PCR reactions out of this range, such as 5 μM to 20 μM of primer pR produced nearly undetectable amounts of products. A two-tube PCR reaction without P1R primer was conducted in parallel and yielded no products (Figure 2B, left two lanes). We also compared the PCR efficiency by varying the annealing temperature (from 50 °C to 60 °C), ratios of template, and number of cycles (from 25 to 35 cycles), but no improvements in performance were observed other than those described above (data not shown).

Next, we introduced these PCR products into competent *E.coli* cells to assess their recombination capacity. As shown in Figure 2C, both unpurified and purified asymmetric PCR products yielded double resistant colonies in (Kan + Amp) LB plates. In comparison, the two-tube PCR reaction products and the mixture of insert and pUC19 (no extension) did not generate recombinants in (Kan + Amp) plates. (Kan + Amp) plates transformed with either pIRES2-EGFP or pUC19 yielded no viable colonies, ruling out the possibility of genomic integration of the kan^R^ cassette into the *E.coli* genome.

Two lines of evidence proved the identity of double-resistant recombinants. First, an artificial ApaI restriction site absent from pIRES2-EGFP and pUC19 was introduced into pF primer. Double digestion of pIRES2-EGFP, pUC19 and (Kan + Amp) plasmid by ApaI and SalI revealed that the 1.6 kb kan^R^ cassette was released from the recombinant (Figure 2D). Second, sequencing demonstrated that the insert had been fused into the pre-selected site in pUC19 as demarcated by the flanking ApaI and SalI sites (Figure 2E). This was found to be the case for all sequenced single colonies (n = 21; data not shown), indicating a 100% recombination efficiency and total fidelity.

By using a double resistance reporter assay and analyzing the inherent properties of single-tube PCR reaction, we have proven the concept of our novel method and consequently refer to this new form of DNA engineering as ABI-REC cloning for asymmetric bridge PCR and intramolecular homologous recombination.

### Effects of homology and insert length on the efficiency of ABI-REC cloning

**Figure 3:**
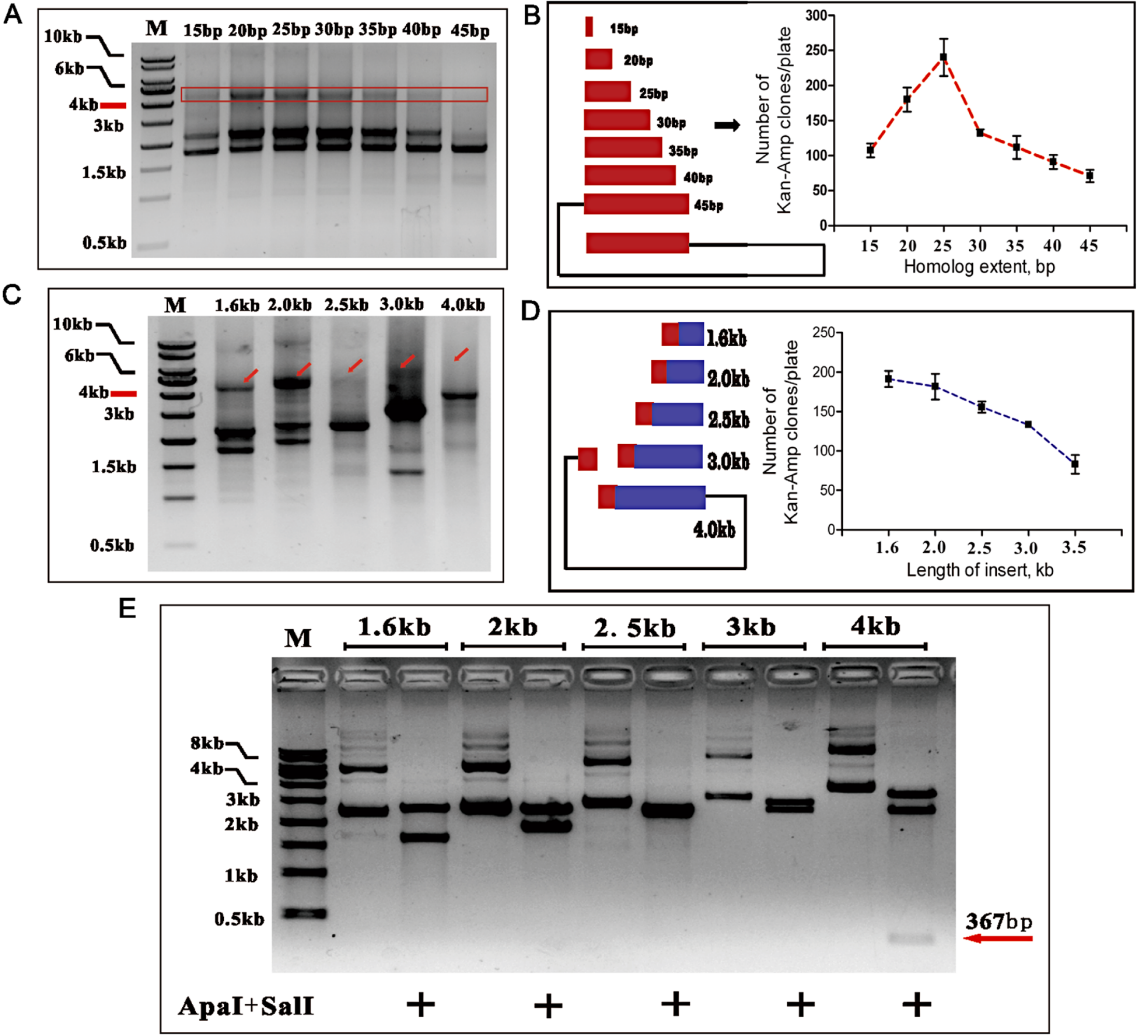
**Effects of homology and insert length upon the efficiency of ABI-REC.** (**A**) Homology was varied by increasing the length of P1R primer in the asymmetric bridge PCR reaction. The reactions with 20 bp and 25 bp P1R primers gave rise to maximum output of fused fragments. (**B**) Transformation of equal volumes of these PCR products into *E.coli* cells produced double resistant colonies with various numbers. Colony capacity varied, with colonies producing maximum target fragment output being more numerous. The number of colonies per plate was plotted against homology. Error bars indicate mean ± SD from three independent assays. (**C**) Inserts ranging from 1.6 kb to 4 kb were fused into pUC19 in asymmetric PCR reactions. Red arrows denote the fused fragments. (**D**) Transformation of equal volumes of these PCR products into *E.coli* cells produced various numbers of double resistant colonies. Quantitation of single colonies revealed that colony capacity varied, with colonies producing maximum target fragment output being more numerous. The number of colonies per plate was plotted against insert size. Error bars indicate mean ± SD from three independent assays. (**E**) ApaI and SalI restriction digestion of the plasmids extracted from the single colonies in (**D**). All plasmids released the inserts as designed. Please note that 2.5 kb insert is nearly identical to backbone pUC19, as one band was observed. In addition, the 4 kb insert has three ApaI sites, one of which is only 133 bp in size and therefore undetectable in the gel. A 367 bp band is denoted by a red arrow.

Next we sought to evaluate the cloning efficiency of ABI-REC by examining two parameters: homology and insert length. The homologous end of primer P1R was altered by decreasing the length from 45mer to 15mer. Asymmetric bridge PCR showed that the amplification efficiency reached a peak around 20 or 25mer. Both longer and shorter primers were found to be less efficient (Figure 3A). The colony formation capacity of equal volumes of these products was consistent with the PCR reaction efficiency (Figure 3B), implying a direct link between PCR output and colony formation. This test also demonstrated that P1R oligos of up to 25 mer in length are optimal for efficient PCR reaction and a high gene fusion ratio.

We then assessed cloning efficiency by fusing selected inserts varying in size from 1.6 kb to 4 kb. A 25 mer P1R primer was used in all reactions. The asymmetric bridge PCR reaction revealed a varied PCR output (Figure 3C). Transformation of these PCR products yielded declining number of recombinant colonies with respect to the insert length (Figure 3D). Although long inserts were more difficult to fuse with the target plasmid, our data demonstrated that this method is applicable to the cloning of DNA sequences up to 4 kb in size. Sequencing (n = 12 for each insert) and restriction digestion revealed that all these recombinants harbored the expected inserts at the pre-selected sites (Figure 3E).

In summary, a 25 mer length of homology, and a shorter insert size produced the highest yield of PCR products from the asymmetric bridge PCR reaction, and consequently, the highest yield of recombinant colonies.

### Construction and expression of porcine myostatin reporters by ABI-REC

To determine whether ABI-REC could be used to clone endogenous DNA sequences intact rather than DNA stretches from plasmids, we next applied it to direct cloning from a complex mammalian genome. The pig myostatin (MSTN) gene was chosen because it has been proven to be a muscle growth inhibitor in mice, cows, sheep and humans [10]. MSTN knockout mice are already known to be viable and fertile [11]. In this regard, MSTN locus has become a safe harbor for transgene expression in animals. We believe that it would be particularly useful to identify porcine MSTN transcriptional regulation elements in order to introduce site-specific modification at this locus for stable transgene expression.

**Figure 4:**
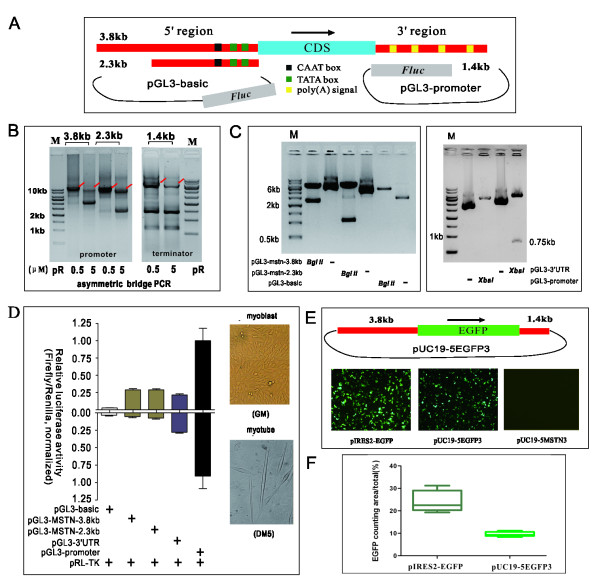
**Identification of porcine MSTN gene regulatory elements by ABI-REC**. (**A**) Genomic structure and reporter design of porcine MSTN gene regulatory elements. Promoter regions 3.8 kb and 2.3 kb in size were fused into a pGL3-basic plasmid, and a 1.4 kb terminator region was fused into a pGL3-promoter plasmid. (**B**) Asymmetric PCR reactions were used to generate the fused fragments of three sequences stated above. Here, template amounts were assessed at 100 ng and 10 ng. Red arrows denote the fused fragment. (**C**) The PCR products in (B) were treated as indicated in “Methods”. Single colonies were selected and sequenced. Digestion mapping indicated that the inserts had been successfully fused into target plasmids. (**D**) The transcriptional activity of the cloned porcine MSTN gene regulatory elements. Equal molar quantities of all these constructs were transfected into mouse myoblast C2C12 cells, and then the luciferase level was measured under either proliferating or differentiating conditions. pRL-TK plasmid that expresses Rluc was co-transfected as internal control. Relative luciferase activity was calculated as ratio over that observed in control transfections, where Fluc activities were normalized to Rluc activities. Error bars indicate mean ± SD from three independent transfections, each in triplicate. The inset indicates proliferating and differentiating C2C12 cells. (**E**) Function of MSTN elements with respect to expression of the reporter gene. The MSTN promoter, EGFP CDS, and MSTN terminator were assembled as an expression cassette by ABI-REC, and their expressivity was assessed by transfecting it into C2C12 cells. This expression cassette is capable of driving EGFP expression efficiently, in comparison to positive control pIRES2-EGFP. Exposure time, 0.5 s; scale, 10 μm. (**F**) Quantitation of EGFP intensity in (E) by ImageJ. Five randomly captured EGFP clips in each transfection were analyzed by ImageJ to calculate the counting area of fluorescent cells as an indicator of EGFP expression level (Green/total × 100%). The threshold was set between 88-225 pixels. (CMV-EGFP-SV40 polyA) cassette of pIRES2-EGFP is the positive control to pig MSTN (promoter-EGFP-terminator) cassette. This result implies that the two identified MSTN elements are able to work coordinately to regulate gene expression. This cassette could be used to control transgene expression and to transfer genes of interest to endogenous sites on the pig MSTN locus.

In line with computational analysis of the pig MSTN gene sequence (Figure 4A), we designed primers to fuse its promoter (3.8 kb and 2.3 kb) and terminator (1.4 kb) into luciferase reporters using ABI-REC. We first determined whether asymmetric bridge PCR would work for non-plasmid DNA templates and amplify long fused sequences. As shown in Figure 4B, the asymmetric PCR reaction robustly produced the expected fused sequences (8.6 kb, 7.1 kb and 6.2 kb, respectively), especially at lower pR primer concentrations. We also explored different reaction parameters, such as the ratio of plasmid to genomic DNA and the number of cycles, but no improvements in performance were observed (data not shown). This was consistent with findings observed in the double resistance reporter assay. These data further demonstrated asymmetric primer concentrations in the bridge PCR to be a rate-limiting step for successful production of fused sequences.

Next, we investigated the time required for DpnI digestion and its role in cloning efficiency. Different from the double-resistance system, a single selectable marker is used in most cases. Therefore, removal of circular plasmid was critical to diminish background. An aliquot of selected reaction products was treated with DpnI for 1 or 2 hours and transformed into competent DH5α cells. As outlined in Table 1, DpnI treatment with 1 and 2 hours had yielded sufficient recombinants carrying inserts of choice with nearly 100% efficiency. The sequencing of randomly selected colonies revealed the insertion at pre-defined sites (data not shown). Single restriction nuclease treatment was carried out to confirm the sequencing result. BglII was used to digest promoter recombinants because of one natural BglII site in the cloned sequences and another in pGL3-basic plasmid. BglII digestion released fragments of expected sizes (1196 bp for 2.3 kb promoter and 2700 bp for 3.8 kb promoter). XbaI was used to digest terminator reporter and released a 0.75 kb fragment as anticipated (Figure 4C). These evidences indicated porcine MSTN gene regulatory elements had been successfully cloned into luciferase reporters by ABI-REC. These constructs were named pGL3-mstn-3.8 kb, pGL3-mstn-2.3 kb, pGL3-mstn-3′UTR. The SV40 polyA signal sequence in pGL3-promoter was replaced by the 1.4 kb terminator sequence, indicating that concerted DNA insertion and deletion (swapping) can be achieved in a single turnaround of ABI-REC cloning.

**Table 1:** Optimized DpnI digestion time for ABI-REC

**Vector**	**Insert**	**DpnI-1 h**			**DpnI-2 h**		
		**Sequenced**	**Confirmed**	**Positive rate**	**Sequenced**	**Confirmed**	**Positive rate**
pGL3-MSTN-3.8 kb	3.8 kb	11	10	91%	10	10	100%
pGL3-MSTN-2.3 kb	2.3 kb	8	8	100%	13	13	100%
pGL3-3′ UTR	1.4 kb	9	8	89%	9	9	100%
Mean cloning efficiency				93%			100%

Third, the activity of these reporters was assessed in mouse myoblast C2C12 cell line because MSTN is dominantly expressed in muscle tissues of animals [12,13]. Equal molar quantities of these plasmids were transfected into C2C12 cells and luciferase activity was measured at selected time points. C2C12 differentiation status was induced with 2% horse serum for 5 days. As shown in Figure 4D3.8 kb and 2.3 kb MSTN promoters had similar activity in both proliferation and differentiation conditions, but it was lower in proliferation than that in differentiation. MSTN terminator had similar activity in both conditions. This experiment indicated that the cloned porcine MSTN gene regulatory elements are functionally active in a mammalian cellular context. These data also imply that the porcine MSTN gene expression is likely to be tightly regulated by its promoter in differentiated muscle cells. This needs to be further investigated by quantifying nascent MSTN mRNA at endogenous sites.

Fourth, we examined the expressivity of porcine MSTN transcriptional unit by constructing a promoter-terminator expression cassette. The MSTN promoter, EGFP coding sequence, MSTN terminator were assembled using ABI-REC (Figure 4E). The expression cassette plasmid was named pUC19-5EGFP3 and empty expression cassette plasmid named pUC19-5MSTN3. Equal molar quantities of these plasmids were transfected into C2C12 cells and green fluorescence was captured by Leica microscope. pUC19-5EGFP3 was able to express EGFP, in comparison to empty plasmid and a positive control pIRES2-EGFP. Quantitation of EGFP fluorescence intensity by ImageJ indicated the expression level of pig MSTN cassette (promoter-EGFP-terminator) to be comparable to that of the constitutive cassette (CMV-EGFP-SV40 polyA) (Figure 4F). We concluded that the identified porcine MSTN gene regulatory elements functioned in synergy to drive the expression of gene of interest.

## Discussion

The reliable and rapid native DNA cloning strategy described here is based on an asymmetric single-tube bridge PCR reaction and intramolecular homologous recombination in *E.coli*. Asymmetric PCR has two major advantages. The first is the introduction of the outermost oligo (primer P1R), which anneals to the end of the linear fragment and so produces large amounts of fused sequence. This manipulation results in an accurate and highly efficient gene fusion process that enables rapid cloning free of unwanted bases. This process can be used for a wide variety of DNA molecules than previously reported PCR-based gene cloning approaches [7,14-17]. The second advantage is that the asymmetric reaction ensures that the insert is amplified in a limited amounts while DNA polymerase generates long fused products. The concentration of pR primer was found to be critical to the success of PCR output and colony formation capacity. This was also shown during the cloning of porcine MSTN sequences. This addresses the key question of how to enhance the efficiency in a PCR-based cloning method. In addition, we compared ABI-REC with a two-step PCR-based cloning protocol described previously [18]. Although it remains unknown why we did not find any singe colonies in the two-step protocol, our work demonstrated that ABI-REC is powerful in term of cloning efficiency (Figure 2).

The double resistance reporter assay is a good way to prove the underlying principles of ABI-REC. This model enabled us to investigate the inherent properties of ABI-REC, including the effects of homology and insert length. In this study, we found 20-25 bp homologous arms to be sufficient to produce long fused sequences in PCR and to provoke efficient recombination in bacteria. These short homologous arms allows ABI-REC to be classified as an oligonucleotide-based strategy. In this way, it is cost-effective. We extended the insert length from 1.6 kb to 4 kb, allowing this process to cover a wide range of genes of interest in molecular biology. We predict that longer DNA sequences can be cloned by ABI-REC, in the assumption that the bridge PCR reaction will become optimized with either potent DNA polymerase or a more robust buffering system. This will definitely be investigated in future studies.

Although the double resistance reporter assay is an ideal proof-of-principle model, it is not very versatile in gene engineering. This is largely because, in many cases, only a single resistance marker is present. This makes it difficult to apply ABI-REC to single-marker cloning systems. In other words, it is important to remove the original circular plasmid to diminish background. DpnI is the method of choice for this process because it is capable of cleaving the methylated nucleotides present in plasmid [19]. We studied the association between time required for DpnI digestion and the ratio of positive colonies. We found that with increasing DpnI digestion time, the positive rate shifted from 93% to 100%. This indicates that ABI-REC is nearly zero-background under single-marker conditions with suitable DpnI treatment. A previous study introduced a new logic gene engineering method based on intermolecular (linear plus circular) homologous recombination in bacteria, but this study failed to address the background of methylated circular plasmids in the single marker system [20]. Another previous study also attempted to enhance mutation efficiency by adding limited amounts of circular plasmid in PCR reaction [21]. ABI-REC outperforms these two methods in that removal of circular plasmid and intramolecular recombination reduce the time required for single marker cloning experiments. Given the ultra-high efficiency of ABI-REC, sequencing is required on a very limited number of clones to check for the presence of junction sites. Please note that a *dam* + *E.coli* strain like DH5α is required for successful ABI-REC in order to discriminate against methylated and hemimethylated parental plasmids.

The two major findings presented in this work will have practical implications for genetic engineering and transgenic biology. First, ABI-REC described here will be particularly favorable to gene functional studies. It has been intensively documented that widely used ligation-dependent cloning methods introduce unwanted bases into target region and that these bases can cause the inclusion of extraneous amino acids or unexpected regulatory sequences [8]. These unwanted bases can compromise the function of protein of interest or even bring misleading results. ABI-REC takes advantage of homologous recombination in bacteria so that native sequences can be fused into pre-selected sites in target plasmid without the involvement of any heterologous nucleotides. This method is free of unwanted base and is totally independent of availability of restriction sites and thus highly desirable to DNA engineering and protein function analysis. Moreover, ABI-REC is likely to be a high-throughput method because of the compatibility of its primer design. Presumably, a series of porcine MSTN gene regulatory elements could be cloned just by changing the site of P2 sequence, without any other modifications upon the whole procedures. In addition, ABI-REC works independent of the availability of restriction sites or even the knowledge of the entire sequence of chosen DNA molecules. This makes it amenable to the construction of DNA library. For example, the pF and pR primers could be used as adaptors to link to target DNA or cDNA, and then the linked molecules could be fused into recipient plasmids. Therefore, the experimental design could be adapted to automated high-throughput applications favoring mass generation and large-scale screening of mutants. This will greatly reduce the time, difficulty and labor required to create recombinant plasmids.

Second, the porcine MSTN expression cassette is of highly importance to site-specific modification in transgenic animals. In this work, the porcine MSTN expression cassette was proven to work in a synergistic manner. This cassette could be used to express gene of interest as commonly used gene expression unit. Genes of interest could be introduced into the native porcine MSTN locus for *in situ* expression through gene targeting or ZFN technology [22]. Taking into account the research conducted in MSTN knockout mice [11], one can conclude that MSTN locus is a safe harbor for genome modification. The present study has identified the docking site for transgene integration. This will be of significance to transgenesis in pigs. Given the high conservation of MSTN across mammals, the gene regulatory elements identified here will also be helpful to the study of other types of transgenic livestocks, such as cattle and sheep. The application of this locus in transgenic large animals is now underway.

## Conclusions

We have developed a novel DNA cloning method named ABI-REC. It mainly featured a 3-primer asymmetric PCR and intramolecular homologous recombination. This new method eliminated the use of restriction site and ligation, excluding operational nucleotides. It does not involve any PCR purification, gel excision and expensive cloning kits. The flexible and formulated primer design allows directional and site-specific positioning of insert into any pre-selected location in any desired plasmid. Using a double-resistance reporter assay and pig MSTN gene regulatory element identification, we have demonstrated its reliability, reproducibility and high efficiency. We anticipate that ABI-REC will be applicable to DNA engineering and gene functional analysis.

## Methods

### Primer design and oligo synthesis

Three primers were used for ABI-REC cloning. Primer pF(1+2) was composed of two parts: The P2 primer was complementary to the insert and the P1 primer was identical to the 5’ sequence of pre-selected insertion site. Primer pR(3+4) consisted of two arms: the P4 arm was complementary to the insert and P3 arm annealed to the 3’ sequence of insertion site. Primer P1R was complementary to primer P1. The annealing temperature in the PCR reaction was 5°C less than the Tm of primer P1R. The sequences of all oligos used in this work were chemically synthesized by Invitrogen (Shanghai) and are listed in Table 2. Note that oligos longer than 40bp were PAGE-purified, whereas those shorter than 40bp were desalted. All oligos were dissolved in TE (pH8.0) buffer and stored at -20°C.

**Table 2:** Primers and oligos used in this study

**Primer**	**Sequence(5′-3′)**	**Comments**
Kan^R^-F	agctatgaccatgattacgGGCccTAGCGGTCACGCTGCGCGTAACC	Underlined is the artificial ApaI (GGGCCC) site; the two primers were for amplifying 1.6 kb kan^R^ cassette from pIRES2-EGFP
Kan^R^-R	gtcgacctgcaggcatgcaagctt CAAACGACCCAACACCGTGCG	
Kan^R^-Right-15	atcatggtcatagct	incremental homolog length from 15 bp to 45 bp
Kan^R^-Right-20	ccgtaatcatggtcatagct	
Kan^R^-Right-25	agggcccgtaatcatggtcatagct	
Kan^R^-Right-30	ccgcta gggcccgtaatcatggtcatagct	
Kan^R^-Right-35	cgtga ccgcta gggcccgtaatcatggtcatagct	
Kan^R^-Right-40	cgcag cgtga ccgcta gggcccgtaatcatggtcatagct	
Kan^R^-Right-45	ttacg cgcag cgtga ccgcta gggcccgtaatcatggtcatagct	
Kan^R^-F-2 kb	agctatgaccatgattacgggccc AACCAATAGGCCGAAATCGGC	incremental insert length
Kan^R^-F-2.5 kb	agctatgaccatgattacgggccc TCGCCGACCACTACCAGCAG	
Kan^R^-F-3 kb	agctatgaccatgattacgggccc ACCGGGGTGGTGCCCATCC	
Kan^R^-F-4 kb	agctatgaccatgattacgggccc GGCATTATGCCCAGTACATGACC	
pF(3.8 kb)	gctcgagatc tgcgatctaa gtaagcttgg CATCATTAAACTTCTGACAAGCC	3.8 kb porcine MSTN promoter region
pF(2.3 kb)	gctcgagatc tgcgatctaa gtaagcttgg GTGCCATGAGTATTGATTCTGGAG	2.3 kb porcine MSTN promoter region
pR(promoter)	catggtggctttaccaacagtaccggaatg CGCCAAGCAAAATTTTAATGCC	porcine MSTN promoter region
P1R(promoter)	ttagatcgcagatctcgagc	3′ outermost primer for promoter
pF(1.4 kb)	gcagacatga taagatacat tgatg GGTTCATTACTTCCTAAAACATGG	porcine MSTN terminator
pR(terminator)	CTCTCAAGGG CATCGGTCGA CGGATCC GTTTCTACACATTAGATGTAAG	
P1R(terminator)	CATCA ATGTATCTTA TCATGTCTGC	3′ outermost primer for terminator
MSTN-P-F	gaattcgagctcggtacccgg CATCATTAAACTTCTGACAAGCC	construction of porcine MSTN expression cassette driving the expression of EGFP
MSTN-P-R	gcaggtcgactctagaggatcc GCCAAGCAAAATTTTAATGCC	
MSTN-P-Right	ccgggtaccgagctcgaattc	
MSTN-T-F	aagcttggcgtaatcatggtc ATTTATATTTGGTTCATTACTTCC	
MSTN-T-R	tttcacacaggaaacagctat CTTACATCTAATGTGTAGAAAC	
MSTN-T-Right	gaccatgattacgccaagctt	
EGFP-F	CATCATTAAACTTCTGACAAGCC ACCATGGTGAGCAAGGGCG	
EGFP-R	GGAAGTAATGAACCAAATATAAAT TTACTTGTACAGCTCGTCCATGCC	
EGFP-Right	GGCTTGTCAGAAGTTTAATGATG	

### Bacterial strains, backbone plasmids and DNA templates

*E.coli* strain DH5α [genotype:F- supE44_lacU169 f80 lacZ_ M15 hsdR17 recA1 0endA1 gyrA96 thi-1 relA1] (Takara, Japan) was used for all DNA cloning assays in this study. It was grown in LB medium (Luria-Bertani), where ampicillin and kanamycin were added at 50μg/ml and 100μg/ml, respectively, when necessary. Exogenous DNA was introduced into competent DH5α cells by chemical transformation. The pUC19 cloning plasmid was from Takara (Japan). pIRES2-EGFP was purchased from Clontech (U.S.). PGL3-basic, pGL3-promoter and pRL-TK luciferase reporters were purchased from Promega (U.S.). All these plasmids were cultured and propagated according to the manufacturers’ specifications and purified using an Ultrapure Plasmid Extraction Kit (Tiangen, Beijing, China). Hubei white pig ear tissue genomic DNA was extracted using a Roche High Pure PCR Template Preparation Kit. All DNAs solutions were measured using a NanoVue^TM^ (GE Healthcare) spectrophotometer to determine purity and concentration.

### Enzymes and reagents

ApaI, SalI, DpnI, BglII and XbaI restriction nucleases, *Taq* DNA polymerase and dNTP were purchased from Fermentas (Lithuania). KOD Plus high-fidelity DNA polymerase was purchased from Toyobo (Japan). A 1kb DNA ladder was from Dongsheng Co. Ltd. (Guangzhou, China). Enzymatic reactions were carried out under recommended conditions. All other chemicals used in the study were of molecular biology grade.

### Asymmetric single-tube bridge PCR reaction system

Asymmetric single-tube bridge PCR was conducted in a 50μl mixture: 10 μM P1P2 1μl (200nM final), 0.1-1 μM P3P4 1 μl (2-20 nM final), 10μM P1R 1 μl (200nM final), DNA template appropriate quantity, 2 mM dNTP 5 μl , 25 mM MgSO_4_ 2 μl, 10×KOD buffer 5 μl, KOD Plus 1 μl (1 unit), PCR-grade water 33μl. For Kan-Amp double-resistance reporter construction, the following DNA template was included: pIRES2-EGFP 50ng and pUC19 100ng. For porcine MSTN reporter vectors the following template was added: Hubei white pig genomic DNA 100ng, pGL3-basic/promoter plasmid 100ng. The PCR conditions were as follows: 95°C 2min, 30 cycles of (95°C for 15s, 55°C for 30s, 68°C for X min; X=total fused plasmid length/1kb). The PCR products were size fractionized by 1% agarose gel electrophoresis and documented by ChemiDoc^™^ XRS system (Bio-Rad, U.S.). As for the two-step PCR protocol, see details in (10).

### PCR product processing, transformation and plasmid construction

The PCR products were digested by DpnI at 37°C for 1 or 2 hours to destroy methylated circular plasmids with the following reaction: PCR products, 26 μl, 10×Tango buffer 3 μl, DpnI 1 μl (1 unit). DpnI endonuclease works well in KOD Plus buffer so we did not typically take extra steps to purify PCR products. Aliquots of 5 μl digested PCR products were chemically transformed into 100 μl competent *E.coli* DH5α to generate recombinants. The Kan-Amp double resistance reporter plasmid was designed as: the 1.6kb kanamycin resistance gene cassette from pIRES2-EGFP was fused into the MCS region of pUC19, and an artificial ApaI restriction site was created to demarcate the insertion site. Porcine MSTN regulatory element reporters were created as follows: 2.3 kb and 3.8 kb porcine myostatin promoter sequences were fused into the upstream of Fluc gene of pGL3-basic reporter, while the 1.4 kb porcine myostatin terminator fragment was fused into the downstream of Fluc gene of pGL3-promoter plasmid, replacing the SV40 late poly(A) signal sequence. For the MSTN expression cassette plasmid, the 3.8 kb promoter, 1.4 kb terminator, and 0.72 kb EGFP cDNA were assembled in that order. PCR products were excised from 1% agarose gel with a scalpel and purified by a purification kit (Generay, Shanghai, China). Purified PCR products were eluted in 30 μl elution buffer (EB) and adjusted to 100 ng/μl. Their size and integrity were confirmed by electrophoresis of 2 μl products. LB plates were analyzed by Quantity One software (Bio-Rad, U.S.) to calculate the number of bacterial colonies. Randomly picked clones were sequenced using a ABI3730 DNA Analyzer (Applied Biosystems, Foster City, CA, U.S.) and the sequences were assembled and analyzed using DNAStar software (DNASTAR Inc., Madison, WI. U.S.).

### Cell culture and transfection

A mouse myoblast C2C12 cell line was cultured in high glucose DMEM (Invitrogen) supplemented with 10% fetal bovine serum (Gibco) at 37°C and 5% CO_2_, saturated humidity. C2C12 differentiation was induced by DMEM with 2% horse serum (Invitrogen). 4×10^4^ cells were seeded in a 24-well plate one day before transfection. For porcine MSTN regulatory element analysis, equal molar quantities of reporter constructs (Fluc served as a reporter) and internal control 250ng pRL-TK (Rluc served as a control) plasmids were co-transfected by Lipofectamine2000 (Invitrogen). Eight hours post transfection, the transfected wells were renewed with fresh medium for further culture. Cells were lysed 48h post transfection under proliferation conditions, or 5 days after differentiation. For MSTN expression cassette assay, equal molar quantities of pIRES2-EGFP, pUC19-5MSTN3 and pUC19-5EGFP3 were transfected into C2C12 cells. Forty-eight hours post transfection, fluorescence was captured using a Leica 4000B microsystem (Germany). All transfections were assessed in three independent experiments, each in triplicate.

### Luciferase assay

A Dual Luciferase Reporter Assay System (Promega) was used to measure luciferase activity in a Glomax luminometer with slight deviations from the manufacturer’s instructions. In brief, 100 μl passive lysis buffer was used to disrupt cells on each well with gentle shaking at room temperature. 10 μl LARII solution was added to 20 μl cell lysate to initiate the primary scanning to measure Fluc luciferase activity. Shortly afterwards, 10 μl substrate solution was added into the same tube to initiate Rluc activity.

### Statistical analysis

The Fluc luciferase activities were normalized to Rluc activities. Luciferase levels were reported as fold repression in activity over that observed in transfections with control treatments. The data represent the mean ± S.D. of three independent experiments and were analyzed by Student’s *t*-test. Differences below *P* < 0.05 were regarded as significant. EGFP fluorescence intensity was analyzed using ImageJ software.

### Accession number

The annotation of these reporters engineered in this work can be retrieved under accession number [Genbank: JN542719, JN542720, JN542721].

## Abbreviations

ABI-REC, Asymmetric bridge PCR and intramolecular homologous recombination; kanR, Kanamycin resistance gene; MSTN, Myostatin; EGFP, Enhanced green fluorescence protein; IRES, Internal ribosome entry site.

## Competing interests

The authors declare that they have no competing interests.

## Authors’ contributions

YB conceived the concept of ABI-REC, designed all primers, designed the double-resistance reporter assay and wrote the manuscript. XZ proposed the pig MSTN locus and designed the functional analysis of it. XQ and ZH optimized asymmetric PCR conditions and helped making the gel image. LZ analyzed all the recombinant plasmids. XL conducted luciferase and EGFP expression analysis. She also analyzed the EGFP intensity by ImageJ. LL prepared the pig genomic DNA. WH cultured mammalian cells. HX performed all cell transfections. JZ and QW performed DNA transformation into *E.coli*. All authors read and approved the manuscript.

## References

[B1] Lu Q Seamless cloning and gene fusion Trends Biotechnol 2005 23 199 207 15780712 10.1016/j.tibtech.2005.02.008 PMC7119129

[B2] Rashtchian A Buchman GW Schuster DM Berninger MS Uracil DNA glycosylase-mediated cloning of polymerase chain reaction-amplified DNA: application to genomic and cDNA cloning Anal Biochem 1992 206 91 97 1456447 10.1016/s0003-2697(05)80015-6

[B3] Smith C Day PJ Walker MR Generation of cohesive ends on PCR products by UDG-mediated excision of dU, and application for cloning into restriction digest-linearized vectors PCR Methods Appl 1993 2 328 332 8324507 10.1101/gr.2.4.328

[B4] Buchholz F Bishop M LoxP-directed cloning: use of Cre recombinase as a universal restriction enzyme Biotechniques 2001 31 906 908 910, 912, 914, 916, 918 11680722 10.2144/01314rr02

[B5] Sleight SC Bartley BA Lieviant JA Sauro HM In-Fusion BioBrick assembly and re-engineering Nucleic Acids Res 2011 38 2624 2636 20385581 10.1093/nar/gkq179 PMC2860134

[B6] Kuzuya A Tanaka K Katada H Komiyama M Restriction enzyme treatment/ligation independent cloning using caged primers for PCR Nucleic Acids Symp Ser 2009 53 75 76 10.1093/nass/nrp038 19749267

[B7] Bryksin AV Matsumura I Overlap extension PCR cloning: a simple and reliable way to create recombinant plasmids Biotechniques 2010 48 463 465 20569222 10.2144/000113418 PMC3121328

[B8] Ohtsuka M Kimura M Tanaka M Inoko H Recombinant DNA technologies for construction of precisely designed transgene constructs Curr Pharm Biotechnol 2009 10 244 251 19199958 10.2174/138920109787315033

[B9] Malureanu LA Targeting vector construction through recombineering Methods Mol Biol 2011 693 181 203 21080281 10.1007/978-1-60761-974-1_11

[B10] Clop A Marcq F Takeda H Pirottin D Tordoir X Bibe B Bouix J Caiment F Elsen JM Eychenne F A mutation creating a potential illegitimate microRNA target site in the myostatin gene affects muscularity in sheep Nat Genet 2006 38 813 818 16751773 10.1038/ng1810

[B11] McPherron AC Lawler AM Lee SJ Regulation of skeletal muscle mass in mice by a new TGF-p superfamily member Nature 1997 387 83 90 9139826 10.1038/387083a0

[B12] Ji S Losinski R Cornelius S Frank G Willis G Gerrard D Depreux F Spurlock M Myostatin expression in porcine tissues: tissue specificity and developmental and postnatal regulation Am J Physiol 1998 275 R1265 R1273 9756559 10.1152/ajpregu.1998.275.4.R1265

[B13] Patruno M Caliaro F Maccatrozzo L Sacchetto R Martinello T Toniolo L Reggiani C Mascarello F Myostatin shows a specific expression pattern in pig skeletal and extraocular muscles during pre- and pos-natal growth Differentiation 2008 76 168 181 17573916 10.1111/j.1432-0436.2007.00189.x

[B14] Chiu J March PE Lee R Tillett D Site-directed, Ligase-Independent Mutagenesis (SLIM): a single-tube methodology approaching 100% efficiency in 4 h Nucleic Acids Res 2004 32 e174 15585660 10.1093/nar/gnh172 PMC535700

[B15] Wu W Jia Z Liu P Xie Z Wei Q A novel PCR strategy for high-efficiency, automated site-directed mutagenesis Nucleic Acids Res 2005 33 e110 16030347 10.1093/nar/gni115 PMC1178011

[B16] Fu C Wehr DR Edwards J Hauge B Rapid one-step recombinational cloning Nucleic Acids Res 2008 36 e54 18424799 10.1093/nar/gkn167 PMC2396420

[B17] Li J Li C Xiao W Yuan D Wan G Ma L Site-directed mutagenesis by combination of homologous recombination and DpnI digestion of the plasmid template in Escherichia coli Anal Biochem 2008 373 389 391 18037368 10.1016/j.ab.2007.10.034

[B18] van den Ent F Lowe J RF cloning: a restriction-free method for inserting target genes into plasmids J Biochem Biophys Methods 2006 67 67 74 16480772 10.1016/j.jbbm.2005.12.008

[B19] Shenoy AR Visweswariah SS Site-directed mutagenesis using a single mutagenic oligonucleotide and DpnI digestion of template DNA Anal Biochem 2003 319 335 336 12871732 10.1016/s0003-2697(03)00286-0

[B20] Zhang Y Buchholz F Muyrers JP Stewart AF A new logic for DNA engineering using recombination in Escherichia coli Nat Genet 1998 20 123 128 9771703 10.1038/2417

[B21] Martin A Toselli E Rosier MF Auffray C Devignes MD Rapid and high efficiency site-directed mutagenesis by improvement of the homologous recombination technique Nucleic Acids Res 1995 23 1642 1643 7784224 10.1093/nar/23.9.1642 PMC306911

[B22] Moehle EA Rock JM Lee YL Jouvenot Y DeKelver RC Gregory PD Urnov FD Holmes MC Targeted gene addition into a specified location in the human genome using designed zinc finger nucleases Proc Nalt Acad Sci USA 2007 104 3055 3060 10.1073/pnas.0611478104 PMC1802009 17360608

